# Changes in the circumscription of *Deprea* (Physalideae, Solanaceae): thirty two new combinations

**DOI:** 10.3897/phytokeys.46.9069

**Published:** 2015-02-27

**Authors:** Rocío Deanna, Segundo Leiva González, Gloria Estela Barboza

**Affiliations:** 1 Instituto Multidisciplinario de Biología Vegetal (IMBIV), CONICET and Universidad Nacional de Córdoba, CC 495, CP 5000, Córdoba, Argentina; 2 Museo de Historia Natural, Universidad Privada Antenor Orrego de Trujillo, CC 1075, Trujillo, Perú; 3 Facultad de Ciencias Químicas, Universidad Nacional de Córdoba, Haya de la Torre y Medina Allende, Córdoba, Argentina

**Keywords:** *Deprea*, combinations, Solanaceae, *Larnax*

## Abstract

According to the latest phylogenetic and cytogenetic results, *Larnax* and *Deprea* should be merged in order to form a natural group. Consequently, we propose 32 combinations of *Larnax* species names under *Deprea*: *Deprea abra-patriciae* (S.Leiva & Barboza) S.Leiva & Deanna, **comb. nov.**, *Deprea altomayoensis* (S.Leiva & Quip.) Barboza & Deanna, **comb. nov.**, *Deprea andersonii* (N.W.Sawyer) Deanna & S.Leiva, **comb. nov.**, *Deprea bongaraensis* (S.Leiva) Deanna & Barboza, **comb. nov.**, *Deprea chotanae* (S.Leiva, Pereyra & Barboza) S.Leiva, **comb. nov.**, *Deprea darcyana* (N.W.Sawyer) Barboza & S.Leiva, **comb. nov.**, *Deprea dilloniana* (S.Leiva, Quip. & N.W.Sawyer) Barboza, **comb. nov.**, *Deprea grandiflora* (N.W.Sawyer & S.Leiva) Deanna & Barboza, **comb. nov.**, *Deprea harlingiana* (Hunz. & Barboza) S.Leiva & Deanna, **comb nov.**, *Deprea hawkesii* (Hunz.) Deanna, **comb. nov.**, *Deprea kann-rasmussenii* (S.Leiva & Quip.) S.Leiva & Barboza, **comb. nov.**, *Deprea longipedunculata* (S.Leiva, E.Rodr. & J.Campos) Barboza, **comb. nov.**, *Deprea lutea* (S.Leiva) Deanna, **comb. nov.**, *Deprea macasiana* (Deanna, S.Leiva & Barboza) Barboza, **comb. nov.**, *Deprea maculatifolia* (E.Rodr. & S.Leiva) S. Leiva, **comb. nov.**, *Deprea nieva* (S.Leiva & N.W.Sawyer) Barboza & Deanna, **comb. nov.**, *Deprea parviflora* (N.W.Sawyer & S.Leiva) S.Leiva, **comb. nov.**, *Deprea pedrazae* (S.Leiva & Barboza) Deanna & S.Leiva, **comb. nov.**, *Deprea peruviana* (Zahlbr.) S.Leiva & Barboza, **comb. nov.**, *Deprea pilosa* (S.Leiva, E.Rodr. & J.Campos) Deanna, **comb. nov.**, *Deprea pomacochaensis* (S.Leiva) Barboza, **comb. nov.**, *Deprea psilophyta* (N.W.Sawyer) S.Leiva & Deanna, **comb. nov.**, *Deprea pumila* (S.Leiva, Barboza & Deanna) S.Leiva, **comb. nov.**, *Deprea purpurea* (S.Leiva) Barboza & S.Leiva, **comb. nov.**, *Deprea purpureocarpa* (S.Leiva, Deanna & Barboza) Deanna, **comb. nov.**, *Deprea sachapapa* (Hunz.) S.Leiva & Deanna, **comb. nov.**, *Deprea sagasteguii* (S.Leiva, Quip. & N. W.Sawyer) Barboza, **comb. nov.**, *Deprea sawyeriana* (S.Leiva, E.Rodr. & J.Campos) S.Leiva, **comb. nov.**, *Deprea schjellerupiae* (S.Leiva & Quip.) Barboza & Deanna, **comb. nov.**, *Deprea steyermarkii* (Hunz.) S.Leiva & Barboza, **comb. nov.**, *Deprea toledoana* (Barboza & S.Leiva) Barboza, **comb. nov.**, and *Deprea vasquezii* (S.Leiva, E.Rodr. & J.Campos) Deanna, **comb. nov.**

## Introduction

*Larnax* Miers and *Deprea* Raf. are two closely related genera of Physalideae tribe (Solanaceae) that include 35 and 10 species, respectively. Both genera are restricted to the neotropics mainly inhabiting pre-montane and montane cloud forest from Bolivia to Costa Rica. Both *Larnax* and *Deprea* include species ranging from herbs to small trees with funnel-shaped to stellate corollas, but always with accrescent fruiting calyces tightly or loosely enclosing the berries (Sawyer 2005, Deanna et al. 2014a). The main centre of diversity for both genera is Peru and Ecuador (Leiva González et al. 2008, Deanna et al. 2014a, Jørgensen and León-Yánez 1999). Only one taxon extends north into Central America [Larnax sylvarum subsp. sylvarum (Standl. & C.V.Morton) N.W.Sawyer, Sawyer 2001] and one species is found at the south of their range in Bolivia [*Larnax subtriflora* (Ruiz & Pav.) Miers, Leiva González et al. 2013].

*Deprea* and *Larnax* are interesting for their chemical and medicinal value (Cardona et al. 2005, Misico et al. 2011, Casero et al. 2015), but also for their controversial circumscription and position within the Physalideae tribe (Olmstead et al. 2008, Särkinen et al. 2013, Carrizo García et al. submitted). Various authors have transferred species between the two genera for many years (Miers 1849, D´Arcy 1973, 1993, Sawyer 2001, Leiva González et al. 2005) and redefined their limits (Hunziker 2001, Sawyer 2005). Sawyer (2005) differentiated *Deprea* and *Larnax* based on six characters (filament base expansion, filament length, filament adnation, anther length, corolla shape, and pollen surface texture) according to morphological cladistic work, although some characters overlap in the two genera. Moreover, Sawyer (1999) obtained ambiguous results when attempting to set apart these genera using DNA sequence data in cladistic analysis, and the most recent phylogenetic analysis of the family (Särkinen et al. 2013) suggests they are not monophyletic.

Here we deal with the new circumscription of the two genera under *Deprea* and the consequent new combinations of *Larnax* based on recent phylogenetic evidence that show *Deprea* and *Larnax* to be monophyletic when their species are merged (100% bootstrap support), where species, as *Larnax sachapapa* Hunz., are resolved nested within a group of *Deprea* species, or vice versa (Carrizo et al. submitted). The phylogenetic evidence is further supported by results from recent extensive field collecting and taxonomic work which have shown that characters utilized to distinguish the two genera are not useful, and increasing number of species are found to have intermediate features between the two genera (see Table 1, Deanna et al. 2014b; Carrizo et al. submitted). Consequently, this paper synonymises *Larnax* under *Deprea*, expands the morphological circumscription of *Deprea*, and transfers the remaining *Larnax* species to *Deprea*, presenting all 32 new combinations, and a list of all currently accepted 45 names in *Deprea*. Type specimens are also cited with their corresponding barcode number if it is available or their sheet number where it is not (HUT, QCA, QCNE, QUSF, and W).

**Table 1.** Characters used to delimit *Larnax* and *Deprea* according to Barboza and Hunziker (1994), Leiva González et al. (2005), and Sawyer (2005), contrasting with type species of each genera and species recently described having intermediate features (marked in bold).

Character	*Deprea orinocensis* *(type species)	*Larnax subtriflora* *(type species)	*Deprea zamorae* Barboza & S.Leiva*	*Larnax pedrazae* S.Leiva & Barboza	*Deprea* sp. nov. (Deanna & Leiva 138)
Corolla shape	Funnel-shaped	Stellate	**Narrowly campanulate**	Stellate	Campanulate
**Ratio corolla lobes/ corolla tube**	0.4–0.9	2.8–3.3	**(1–) 1.2–1.5**	**1–1.2**	**0.8–1**
Filament length**	Equal	Equal	**Equal or slightly unequal**	Equal	Equal
Filament base expansion**	Gradually expanded	Gradually expanded	**Abruptly expanded**	Abruptly expanded	Abruptly expanded
**Filament adnation**	4.1±0.7	1.3±0.3	**1.7±0.2**	1.5±0.2	1±0.2
**Anther length**	Equal	Subequal	Equal	**Slightly unequal**	Subequal
Mucron in anthers**	Absent	Absent	Absent	Absent	Present
Annular ring of hairs on the inner corolla**	Present	Present	Present	Absent	Absent
Pollen surface texture	Unknown	Dense, minutely microechinate	Widely sparse, minutely microechinate	Unknown	Unknown

* species included in the phylogenetic analysis (Carrizo et al. submitted)

** characters which overlap in type species

## Systematics

### Deprea

Taxon classificationPlantaeSolanalesSolanaceae

Raf., Sylva Tellur. 57. 1838.

Larnax Miers, Ann. Mag. Nat. Hist., ser. 2 (4): 37. 1849, **syn. nov.**

#### Type.

*Deprea orinocensis* (Kunth) Raf. (Lectotype species, designated in D’Arcy 1973 [1974]. Flora of Panama, Part IX. Family 170. Solanaceae. Ann. Missouri Bot. Gard. 60 (3): 624).

Erect shrubs to small trees with spreading branches, exceptionally herbs with axillary inflorescences generally with three to 15 flowers per node, calyx lobes minute or short, exceptionally long and narrowly triangular, corolla funnel-shaped to stellate, stamen petalum broadening gradually to abruptly in width basipetally, with or without auricles, anthers dorsifixed, generally exserted and mucronate, ovary glabrous, fruiting calyx always accrescent, enveloping the fleshy berry tightly or loosely.

### Deprea abra-patriciae

Taxon classificationPlantaeSolanalesSolanaceae

(S.Leiva & Barboza) S.Leiva & Deanna comb. nov.

urn:lsid:ipni.org:names:77145416-1

Larnax abra-patriciae S.Leiva & Barboza, Arnaldoa 16(1): 30. 2009. Basionym

#### Type.

PERU. Amazonas: Bongará, Área de Conservación Privada Abra Patricia-Alto Nieva, km 364–365, carretera Fernando Belaunde Terry, 2250 m, 5°41'28.2"S, 77°48'41.1"W, 16 May 2009 (fl, fr), *S.Leiva 4561* (lectotype, designated in Deanna et al. 2014a, pg. 19: Leiva González & Barboza, Arnaldoa 16(1): 32. Fig. 1. 2009).

### Deprea altomayoensis

Taxon classificationPlantaeSolanalesSolanaceae

(S.Leiva & Quip.) Barboza & Deanna comb. nov.

urn:lsid:ipni.org:names:77145417-1

Larnax altomayoensis S.Leiva & Quip., Arnaldoa 15 (2): 199. 2009 [2008 publ. Jan 2009]. Basionym

#### Type.

PERU. San Martin: Rioja, Bosque de Protección de Alto Mayo, 1010 m, 5°40'11.5"S, 77°37'48.5"W, 24 July 2008 (fl, fr), *S.Leiva, M.Zapata & V.Quipuscoa 4480* (lectotype, designated in Deanna et al. 2014a, pg. 21: Leiva González, Pereyra Villanueva & Barboza, Arnaldoa 15(2): 200. Fig. 1. 2008).

### Deprea andersonii

Taxon classificationPlantaeSolanalesSolanaceae

(N.W.Sawyer) Deanna & S.Leiva comb. nov.

urn:lsid:ipni.org:names:77145432-1

Larnax andersonii N.W.Sawyer, Novon 8 (1): 72. 1998. Basionym

#### Type.

ECUADOR. Napo: km 25 of Hollín-Loreto road, finca entrance next to bridge over a quebrada in secondary pluvial forest, 950 m, 00°40'S, 77°40'W, 1 July 1995 (fl, fr), *N.W.Sawyer & M.Tirado 714* (holotype: MO n.v.; isotypes: CONN! [00054622], QCNE! [105520], US n.v.).

### Deprea bongaraensis

Taxon classificationPlantaeSolanalesSolanaceae

(S.Leiva) Deanna & Barboza comb. nov.

urn:lsid:ipni.org:names:77145418-1

Larnax bongaraensis S.Leiva, Arnaldoa 13 (2): 292. 2006. Basionym

#### Type.

PERU. Amazonas: Bongará, km 328–329, carretera Pomacochas-Nuevo Cajamarca, arriba de laguna Pomacochas, 2400 m, 5 Feb 2006 (fl, fr), *S.Leiva 3543* (lectotype, designated in Deanna et al. 2014a, pg. 21: Leiva González & Rodríguez Rodríguez, Arnaldoa 13(2): 293. Fig. 1. 2006).

### Deprea chotanae

Taxon classificationPlantaeSolanalesSolanaceae

(S.Leiva, Pereyra & Barboza) S.Leiva comb. nov.

urn:lsid:ipni.org:names:77145419-1

Larnax chotanae S.Leiva, Pereyra & Barboza, Arnaldoa 15 (2): 203. 2008. Basionym

#### Type.

PERU. Cajamarca: Chota, Bosque El Pargo, 3050 m, 30 Sep 2004 (fl, fr), *S.Leiva & J.Guevara 2883* (lectotype, designated in Deanna et al. 2014a, pg. 21: Leiva González, Pereyra Villanueva & Barboza, Arnaldoa 15(2): 204. Fig. 3. 2008).

### Deprea darcyana

Taxon classificationPlantaeSolanalesSolanaceae

(N.W. Sawyer) Barboza & S.Leiva comb. nov.

urn:lsid:ipni.org:names:77145433-1

Larnax darcyana N.W.Sawyer, Novon 11 (4): 463. 2001. Basionym

#### Type.

COLOMBIA. Huila: forest around Mehrenberg, road from Popayan, 2350 m, 6 July 1984 (fl, fr), *W.G.D´Arcy, A.Gentry, M.Monsalve & P.Silverstone 15626* (holotype: MO n.v.; isotype: CONN! [00054625]).

### Deprea dilloniana

Taxon classificationPlantaeSolanalesSolanaceae

(S.Leiva, Quip. & N.W.Sawyer) Barboza comb. nov.

urn:lsid:ipni.org:names:77145420-1

Larnax dilloniana S.Leiva, Quip. & N.W.Sawyer, Arnaldoa 5 (1): 85. 1998. Basionym

#### Type.

PERU. San Martín: Rioja, arriba del poblado Miraflores (ca. Nueva Cajamarca), 1260–1420 m, 3 Nov 1996 (fl, fr), *S.Leiva, M.Dillon, I.Sánchez, V.Quipuscoa & P.Lezama 1919* (lectotype, designated in Deanna et al. 2014a, pg. 21: CONN! [00066074]; isolectotypes: CONN! [00066075, 00168924!], CORD! [00004043], F! [2183234], HUT! [031930], MO! [05097641]).

### Deprea grandiflora

Taxon classificationPlantaeSolanalesSolanaceae

(N.W.Sawyer & S.Leiva) Deanna & Barboza comb. nov.

urn:lsid:ipni.org:names:77145434-1

Larnax grandiflora N.W.Sawyer & S.Leiva, Novon 11 (4): 463. 2001. Basionym

#### Type.

PERU. Cajamarca: San Ignacio, in forest along road from San Martín to El Chaupe, 1700 m, 05°11'S, 79°03'W, 26 June 1997 (fl, fr), *N.W.Sawyer 827* (holotype: NY!; isotypes: CONN! [00054628], MO n.v.).

### Deprea harlingiana

Taxon classificationPlantaeSolanalesSolanaceae

(Hunz. & Barboza) S.Leiva & Deanna comb. nov.

urn:lsid:ipni.org:names:77145435-1

Larnax harlingiana Hunz. & Barboza, Kurtziana 24: 157. 1995. Basionym

#### Type.

ECUADOR. Zamora-Chinchipe: Nudo de Sabanilla, E slope c. 5 km from pass on road Yanganá-Valladolid, 2700 m, 4 Apr 1985 (fl, fr), *G.Harling & L.Andersson 23646* (holotype: GB! [0008131]; isotypes: CORD! [00006738], QCA! [156713]).

### Deprea hawkesii

Taxon classificationPlantaeSolanalesSolanaceae

(Hunz.) Deanna comb. nov.

urn:lsid:ipni.org:names:77145436-1

Larnax hawkesii Hunz., Kurtziana 10: 9. 1977. Basionym

#### Type.

COLOMBIA. Huila: Carretera a La Plata, región de Moscopán, Santa Leticia, 2230 m, 21 July 1948 (fl, fr), *H.García-Barriga & J.C.Hawkes 12919* (holotype: US! [00385927]; isotypes: COL! [000004223, 000004224]).

### Deprea kann-rasmussenii

Taxon classificationPlantaeSolanalesSolanaceae

(S.Leiva & Quip.) S.Leiva & Barboza comb. nov.

urn:lsid:ipni.org:names:77145437-1

Larnax kann-rasmussenii S.Leiva & Quip., Arnaldoa 9 (1): 29. 2002 [2003]. Basionym

#### Type.

PERU. San Martín: Huallaga, entre La Ribera y Añazco Pueblo, 1850 m, 1 Sep 2000 (fl, fr), 06°84.705'S 77°48.440'W, *S.Leiva & V.Quipuscoa 2470* (lectotype, designated in Deanna et al. 2014a, pg. 23: HUT! [40031, two sheets]).

### Deprea longipedunculata

Taxon classificationPlantaeSolanalesSolanaceae

(S.Leiva, E.Rodr. & J.Campos) Barboza comb. nov.

urn:lsid:ipni.org:names:77145438-1

Larnax longipedunculata S.Leiva, E.Rodr. & J.Campos, Arnaldoa 5 (2): 194. 1998. Basionym

#### Type.

PERU. Cajamarca: San Ignacio, Caserío La Bermeja, bosques de neblina La Bermeja, Dist. Tabaconas, 1830 m, 4 Jan 1998 (fl, fr), *S.Leiva, J.Campos & E.Rodríguez Rodríguez 2098* (lectotype, designated in Deanna et al. 2014a, pg. 25: CONN! [00055675]; isolectotypes: CORD! [00004044], F! [2198658], HUT! [031885], MO! [04906479]).

### Deprea lutea

Taxon classificationPlantaeSolanalesSolanaceae

(S.Leiva) Deanna comb. nov.

urn:lsid:ipni.org:names:77145439-1

Larnax lutea S.Leiva, Arnaldoa 4 (1): 19. 1996. Basionym

#### Type.

PERU. Cajamarca: Chota, 1 km del poblado de Paraguay (Querocoto-La Granja), 2250 m, 7 Aug 1994 (fl, fr), *S.Leiva González, P.Chuna & J.Cadle 1385* (lectotype, designated in Deanna et al. 2014a, pg. 25: CORD! [00004045]; isolectotype: F! [2177616]).

### Deprea macasiana

Taxon classificationPlantaeSolanalesSolanaceae

(Deanna, S.Leiva & Barboza) Barboza comb. nov.

urn:lsid:ipni.org:names:77145421-1

Larnax macasiana Deanna, S.Leiva & Barboza, Phytotaxa 167 (1): 3. 2014. Basionym

#### Type.

ECUADOR. Morona Santiago: Macas, Cerro San José del Quílamo, 500 m antes de la Virgen Purísima de Macas en el Quílamo, 1369 m, 78°08'19.3"W 02°17'45.4"S, 23 Jan 2013 (fl, fr), *R.Deanna & S.Leiva 111* (holotype: QUSF! [29472]; isotypes: CORD! [00006797, 00006799], HAO!, QUSF! [29480]).

### Deprea maculatifolia

Taxon classificationPlantaeSolanalesSolanaceae

(E.Rodr. & S.Leiva) S.Leiva comb. nov.

urn:lsid:ipni.org:names:77145422-1

Larnax maculatifolia E.Rodr. & S.Leiva, Arnaldoa 9 (2): 102. 2003. Basionym

#### Type.

PERU. Amazonas: Bagua, Imaza, Comunidad Aguaruna de Yamayakat, 390 m, 10 Jan 2001 (fl, fr), *E.Rodríguez Rodríguez, S.Leiva & R.Apanu 2384* (holotype: HUT! [38027]; isotypes: MO! [5763266], USM! [000899]).

### Deprea nieva

Taxon classificationPlantaeSolanalesSolanaceae

(S.Leiva & N.W.Sawyer) Barboza & Deanna comb. nov.

urn:lsid:ipni.org:names:77145440-1

Larnax nieva S.Leiva & N.W.Sawyer, Arnaldoa 10 (1): 106. 2003. Basionym

#### Type.

PERU. Amazonas: Bongará, km 384 carretera Nueva Cajamarca-Pomacochas (Florida), 2000 m, 05.41 S, 77.46 W, 12 June 1997 (fl, fr), *S.Leiva & N.W.Sawyer 2045* (lectotype, designated in Deanna et al. 2014a, pg. 25: Leiva González & Barboza, Arnaldoa 16(1): 32. Fig. 1. 2009).

### Deprea parviflora

Taxon classificationPlantaeSolanalesSolanaceae

(N.W.Sawyer & S.Leiva) S.Leiva comb. nov.

urn:lsid:ipni.org:names:77145441-1

Larnax parviflora N.W.Sawyer & S.Leiva, Novon 11 (4): 466. 2001. Basionym

#### Type.

PERU. Cajamarca: Cutervo, Bosque Cutervo, Parque Nacional Cutervo, NW border of Cordillera Tarros, Chorro Blanco sector, ca. 10 km WNW of San Andres de Cutervo, ca. 2650 m, 6°12'S, 78°46'W, 4 Nov 1990 (fl), *M.O.Dillon, I.Sanchez V. & J.Guevara B. 6141* (holotype: F! [2059067]; isotype: CONN! [00168928]).

### Deprea pedrazae

Taxon classificationPlantaeSolanalesSolanaceae

(S.Leiva & Barboza) Deanna & S.Leiva comb. nov.

urn:lsid:ipni.org:names:77145423-1

Larnax pedrazae S.Leiva & Barboza, Arnaldoa 16 (2): 14. 2009. Basionym

#### Type.

PERU. Amazonas: Bagua, La Peca, Puente El Arenal (ruta La Peca-El Arenal), 1170 m, 5°35'27.2"S, 78°24'20.8"W, 12 Oct 2009 (fl, fr), *S.Leiva, M.Zapata & G.Gayoso 4579* (lectotype, designated in Deanna et al. 2014a, pg. 25: Leiva González, Bravi & Barboza, Arnaldoa 16(2): 16. Fig. 1. 2009).

### Deprea peruviana

Taxon classificationPlantaeSolanalesSolanaceae

(Zahlbr.) S.Leiva & Barboza comb. nov.

urn:lsid:ipni.org:names:77145424-1

Athenaea peruviana Zahlbr., Ann. K. K. Naturhist. Hofmus. 7 (1–2): 7–8. 1892. *Larnax peruviana* (Zahlbr.) Hunz., Kurtziana 10: 9. 1977. Basionym

#### Type.

[PERU. Cajamarca: Cutervo, La Ramada]. Tambillo, 29 July 1878 (fl, fr), *C. de Jelski 54* (lectotype, designated in Deanna et al. 2014a, pg. 25: W! [1891-0004186]).

### Deprea pilosa

Taxon classificationPlantaeSolanalesSolanaceae

(S.Leiva, E.Rodr. & J.Campos) Deanna comb. nov.

urn:lsid:ipni.org:names:77145442-1

Larnax pilosa S.Leiva, E.Rodr. & J.Campos, Arnaldoa 5 (2): 200. 1998. Basionym

#### Type.

PERU. Cajamarca: San Ignacio, San José de Lourdes, Estrella del Oriente, borde de camino, 1600 m, 8 Jan 1998 (fl, fr), *S. Leiva, J. Campos & E. Rodríguez 2108* (lectotype, designated in Deanna et al. 2014a, pg. 26: CONN! [00051759]; isolectotypes: CORD! [00004047], F! [2198655], HUT! [031894], M! [M-0171580], MO! [04908631], NY! [00328792], USM! [000900]).

### Deprea pomacochaensis

Taxon classificationPlantaeSolanalesSolanaceae

(S.Leiva) Barboza comb. nov.

urn:lsid:ipni.org:names:77145425-1

Larnax pomacochaensis S.Leiva, Arnaldoa 13 (2): 299. 2006. Basionym

#### Type.

PERU: Amazonas: Bongará, km 328–329 carretera Bongará-Nueva Cajamarca, arriba de laguna Pomacochas, 2400 m, 5 Feb 2006 (fl, fr), *S.Leiva 3542* (lectotype, designated in Deanna et al. 2014a, pg. 28: Leiva González & Rodríguez Rodríguez, Arnaldoa 13(2): 300. Fig. 5. 2006).

### Deprea psilophyta

Taxon classificationPlantaeSolanalesSolanaceae

(N.W.Sawyer) S.Leiva & Deanna comb. nov.

urn:lsid:ipni.org:names:77145443-1

Larnax psilophyta N.W.Sawyer, Novon 8 (1): 74. 1998. Basionym

#### Type.

ECUADOR. Zamora-Chinchipe: Nudo de Sabanilla, pass on road from Yanganá to Valladolid, elfin forest and clearings, 2800–2900 m, 5 Apr 1985 (fl, fr), *G.W.Harling & L.Andersson 23724* (holotype: NY! [00312927]; isotype: QCA! [95-13/83]).

### Deprea pumila

Taxon classificationPlantaeSolanalesSolanaceae

(S.Leiva, Barboza & Deanna) S.Leiva comb. nov.

urn:lsid:ipni.org:names:77145426-1

Larnax pumila S.Leiva, Barboza & Deanna, Phytotaxa 167 (1): 8. 2014. Basionym

#### Type.

ECUADOR. Pastaza: Mera, rumbo hacia Río Anzú, sendero, 1340 m, 78°04'01.5"W, 01°25'31.6"S, 13 Nov 2011 (fl, fr), *C.I.Orozco, G.E.Barboza, A.Orejuela & S.Leiva 3890* (holotype: CORD! [0006758]; isotypes: COL!, QCA!).

### Deprea purpurea

Taxon classificationPlantaeSolanalesSolanaceae

(S.Leiva) Barboza & S.Leiva comb. nov.

urn:lsid:ipni.org:names:77145444-1

Larnax purpurea S.Leiva, Arnaldoa 4 (1): 16. 1996. Basionym

#### Type.

PERU. Cajamarca: San Ignacio, Ruta San Ignacio-El Chaupe, riachuelo, entre abundantes arbustos, 1510 m, 4 Jan 1995 (fl, fr), *S.Leiva G., P.Lezama A. & P.Chuna 1560* (lectotype, designated in Deanna et al. 2014a, pg. 29: CORD! [00004049]; isolectotypes: F! [2177617], HUT! [031931], MO! [05077371], NY! [00076787], USM! [000901]).

### Deprea purpureocarpa

Taxon classificationPlantaeSolanalesSolanaceae

(S.Leiva, Deanna & Barboza) Deanna comb. nov.

urn:lsid:ipni.org:names:77145427-1

Larnax purpureocarpa S.Leiva, Deanna & Barboza, Phytotaxa 167 (1): 12. 2014. Basionym

#### Type.

ECUADOR. Napo: carretera Cosanga-Baeza, 5.4 km al sur de Baeza, en borde de carretera, 1855 m, 77°52'20.2"W, 00°28'34.2"S, 25 Jan 2013 (fl, fr), *R.Deanna & S.Leiva 125* (holotype: QCNE! [0233491]; isotypes: CORD! [00006800], HAO!).

### Deprea sachapapa

Taxon classificationPlantaeSolanalesSolanaceae

(Hunz.) S.Leiva & Deanna comb. nov.

urn:lsid:ipni.org:names:77145445-1

Larnax sachapapa Hunz., Kurtziana 10: 13–15. 1977. Basionym

#### Type.

ECUADOR. Azuay: between Cruz Pamba and Loma de Canela, in region of Río Sadracay, tributary of Río Mehuir, north of Molleturo, in Cinchona forest, 2315–2500 m, 12 June 1943 (fl, fr), *J.A.Steyermark 52965* (holotype: VEN! [119697]; isotypes: CORD! [00006739], F! [1276519]).

### Deprea sagasteguii

Taxon classificationPlantaeSolanalesSolanaceae

(S.Leiva, Quip. & N.W.Sawyer) Barboza comb. nov.

urn:lsid:ipni.org:names:77145428-1

Larnax sagasteguii S.Leiva, Quip. & N.W.Sawyer, Arnaldoa 5 (1): 86–89. 1998. Basionym

#### Type.

PERU. Piura: Ayabaca, Cerro Aypate, 2800–2880 m, 4°42.94'S, 79°34.25'W, 23 May 1996 (fl, fr), *V.Quipuscoa S., O.Angulo Z. & R.Yahuana 601* (lectotype designated in Deanna et al. 2014a, pg. 30: HUT! [031934]).

### Deprea sawyeriana

Taxon classificationPlantaeSolanalesSolanaceae

(S.Leiva, E.Rodr. & J.Campos) S. Leiva comb. nov.

urn:lsid:ipni.org:names:77145446-1

Larnax sawyeriana S.Leiva, E.Rodr. & J.Campos, Arnaldoa 5 (2): 203. 1998. Basionym

#### Type.

PERU. Cajamarca: San Ignacio, Caserío La Bermeja, bosques de neblina La Bermeja, Dist. Tabaconas, 1830 m, 4 Jan 1998 (fl, fr), *S.Leiva, J.Campos & E.Rodríguez 2097* (lectotype, designated in Deanna et al. 2014a, pg. 31: HUT! [031884]; isolectotypes: CONN! [00055668], CORD! [00004051], F! [2198659], MO! [04906480]).

### Deprea schjellerupiae

Taxon classificationPlantaeSolanalesSolanaceae

(S.Leiva & Quip.) Barboza & Deanna comb. nov.

urn:lsid:ipni.org:names:77145447-1

Larnax schjellerupiae S.Leiva & Quip., Arnaldoa 9 (1): 32. 2002 [2003]. Basionym

#### Type.

PERU. San Martin: Huallaga, La Fila, entre Añazco Pueblo y Leguía, 2000 m, 10 Sep 2000 (fr), *S.Leiva & V.Quipuscoa 2479* (lectotype, designated in Deanna et al. 2014a, pg. 31: HUT! [40030]).

### Deprea steyermarkii

Taxon classificationPlantaeSolanalesSolanaceae

(Hunz.) S.Leiva & Barboza comb. nov.

urn:lsid:ipni.org:names:77145448-1

Larnax steyermarkii Hunz., Kurtziana 10: 11. 1977. Basionym

#### Type.

ECUADOR. Zamora: Arenillas, at junction of río Santa Bárbara and río Tintas, between Ca mpanas and Arenillas, southeast of El Pan, 2195 m, 13 July 1943 (fl, fr), *J.A.Steyermark 53535* (holotype: VEN! [119698]; isotypes: CORD!, F! [1276523, 1739588]).

### Deprea toledoana

Taxon classificationPlantaeSolanalesSolanaceae

(Barboza & S.Leiva) Barboza comb. nov.

urn:lsid:ipni.org:names:77145429-1

Larnax toledoana Barboza & S.Leiva, Phytotaxa 167 (1): 16. 2014. Basionym

#### Type.

ECUADOR. Zamora-Chinchipe: [Cerro Toledo], rumbo a Valladolid desde Yanganá, al costado de la ruta, 2690 m, 79°08'52.7"W, 04°26'30.7°S, 16 Nov 2011 (fl, fr), *C.I.Orozco, G.E.Barboza, A.Orejuela & S.Leiva 3936* (holotype: QCA!; isotypes: CORD! [00006769, 00006770, 00006771], COL!, HAO!).

### Deprea vasquezii

Taxon classificationPlantaeSolanalesSolanaceae

(S.Leiva, E.Rodr. & J.Campos) Deanna comb. nov.

urn:lsid:ipni.org:names:77145449-1

Larnax vasquezii S.Leiva, E.Rodr. & J.Campos, Arnaldoa 5 (2): 206. 1998. Basionym

#### Type.

PERU. Cajamarca: San Ignacio, Dist. San José de Lourdes, Estrella del Oriente, 1600 m, 04°50'S, 78°55'W, 8 Jan 1998 (fl, fr), *S.Leiva, J.Campos & E.Rodríguez 2109* (lectotype, designated in Deanna et al. 2014a, pg. 31: US! [00623565]; isolectotypes: CONN! [00055674], CORD! [00004050], F! [2198657], M! [M-0171574], MO! [04908632], NY! [00328793], US! [00623565], USM! [000902]).

### After these new combinations, the genus *Deprea* includes the following 45 currently accepted species:

1. *Deprea abra-patriciae* (S.Leiva & Barboza) S.Leiva & Deanna

2. *Deprea altomayoensis* (S.Leiva & Quip.) Barboza & Deanna

3. *Deprea andersonii* (N.W.Sawyer) Deanna & S.Leiva

4. *Deprea bitteriana* (Werderm.) N.W.Sawyer & Benítez

5. *Deprea bongaraensis* (S.Leiva) Deanna & Barboza

6. *Deprea cardenasiana* Hunz.

7. *Deprea chotanae* (S.Leiva, Pereyra & Barboza) S.Leiva

8. *Deprea cuyacensis* (N.W.Sawyer & S.Leiva) S.Leiva & Lezama

9. *Deprea cyanocarpa* J.Garzon & C.I.Orozco

10. *Deprea darcyana* (N.W.Sawyer) Barboza & S.Leiva

11. *Deprea dilloniana* (S.Leiva, Quip. & N.W.Sawyer) Barboza

12. *Deprea ecuatoriana* Hunz. & Barboza

13. *Deprea glabra* (Standl.) Hunz.

14. *Deprea grandiflora* (N.W.Sawyer & S.Leiva) Deanna & Barboza

15. *Deprea harlingiana* (Hunz. & Barboza) S.Leiva & Deanna

16. *Deprea hawkesii* (Hunz.) Deanna

17. *Deprea kann-rasmussenii* (S.Leiva & Quip.) S.Leiva & Barboza

18. *Deprea longipedunculata* (S.Leiva, E.Rodr. & J.Campos) Barboza

19. *Deprea lutea* (S.Leiva) Deanna

20. *Deprea macasiana* (Deanna, S.Leiva & Barboza) Barboza

21. *Deprea maculatifolia* (E.Rodr. & S.Leiva) S.Leiva

22. *Deprea nieva* (S.Leiva & N.W.Sawyer) Barboza & Deanna

23. *Deprea nubicola* N.W.Sawyer

24. *Deprea orinocensis* (Kunth) Raf.

25. *Deprea oxapampensis* M.Cueva & I.Treviño

26. *Deprea paneroi* Benítez & M.Martínez

27. *Deprea parviflora* (N.W.Sawyer & S.Leiva) S.Leiva

28. *Deprea pedrazae* (S.Leiva & Barboza) Deanna & S.Leiva

29. *Deprea peruviana* (Zahlbr.) S.Leiva & Barboza

30. *Deprea pilosa* (S.Leiva, E.Rodr. & J.Campos) Deanna

31. *Deprea pomacochaensis* (S.Leiva) Barboza

32. *Deprea psilophyta* (N.W.Sawyer) S.Leiva & Deanna

33. *Deprea pumila* (S.Leiva, Barboza & Deanna) S.Leiva

34. *Deprea purpurea* (S.Leiva) Barboza & S.Leiva

35. *Deprea purpureocarpa* (S.Leiva, Deanna & Barboza) Deanna

36. *Deprea sachapapa* (Hunz.) S.Leiva & Deanna

37. *Deprea sagasteguii* (S.Leiva, Quip. & N.W.Sawyer) Barboza

38. *Deprea sawyeriana* (S.Leiva, E.Rodr. & J.Campos) S. Leiva

39. *Deprea schjellerupiae* (S.Leiva & Quip.) Barboza & Deanna

40. *Deprea steyermarkii* (Hunz.) S.Leiva & Barboza

41. *Deprea subtriflora* (Ruiz & Pav.) D’Arcy

42a. Deprea sylvarum (Standl. & C. V. Morton) Hunz. subsp. sylvarum

42b. Deprea sylvarum (Standl. & C. V. Morton) Hunz. subsp. novogranatensis N.W.Sawyer

43. *Deprea toledoana* (Barboza & S.Leiva) Barboza

44. *Deprea vasquezii* (S.Leiva, E.Rodr. & J.Campos) Deanna

45. *Deprea zamorae* Barboza & S.Leiva

## Supplementary Material

XML Treatment for Deprea

XML Treatment for Deprea abra-patriciae

XML Treatment for Deprea altomayoensis

XML Treatment for Deprea andersonii

XML Treatment for Deprea bongaraensis

XML Treatment for Deprea chotanae

XML Treatment for Deprea darcyana

XML Treatment for Deprea dilloniana

XML Treatment for Deprea grandiflora

XML Treatment for Deprea harlingiana

XML Treatment for Deprea hawkesii

XML Treatment for Deprea kann-rasmussenii

XML Treatment for Deprea longipedunculata

XML Treatment for Deprea lutea

XML Treatment for Deprea macasiana

XML Treatment for Deprea maculatifolia

XML Treatment for Deprea nieva

XML Treatment for Deprea parviflora

XML Treatment for Deprea pedrazae

XML Treatment for Deprea peruviana

XML Treatment for Deprea pilosa

XML Treatment for Deprea pomacochaensis

XML Treatment for Deprea psilophyta

XML Treatment for Deprea pumila

XML Treatment for Deprea purpurea

XML Treatment for Deprea purpureocarpa

XML Treatment for Deprea sachapapa

XML Treatment for Deprea sagasteguii

XML Treatment for Deprea sawyeriana

XML Treatment for Deprea schjellerupiae

XML Treatment for Deprea steyermarkii

XML Treatment for Deprea toledoana

XML Treatment for Deprea vasquezii
